# How location and cellular signaling combine to activate the NLRP3 inflammasome

**DOI:** 10.1038/s41423-022-00922-w

**Published:** 2022-09-20

**Authors:** Anil Akbal, Alesja Dernst, Marta Lovotti, Matthew S. J. Mangan, Róisín M. McManus, Eicke Latz

**Affiliations:** 1 Institute of Innate Immunity, University Hospital Bonn, University of Bonn, 53127 Bonn, Germany; 2 German Center for Neurodegenerative Diseases, 53127 Bonn, Germany; 3 Department of Infectious Diseases & Immunology, UMass Medical School, Worcester, MA 01605 USA; 4 Centre of Molecular Inflammation Research, Norwegian University of Science and Technology, 7491 Trondheim, Norway

**Keywords:** NLRP3, Inflammasome, Mechanism, Localization, Structure, Regulation, Monocytes and macrophages, Immune cell death, NOD-like receptors, Cryoelectron microscopy

## Abstract

NOD-, LRR-, and pyrin domain-containing 3 (NLRP3) is a cytosolic innate immune sensor of cellular stress signals, triggered by infection and sterile inflammation. Upon detection of an activating stimulus, NLRP3 transitions from an inactive homo-oligomeric multimer into an active multimeric inflammasome, which promotes the helical oligomeric assembly of the adaptor molecule ASC. ASC oligomers provide a platform for caspase-1 activation, leading to the proteolytic cleavage and activation of proinflammatory cytokines in the IL-1 family and gasdermin D, which can induce a lytic form of cell death. Recent studies investigating both the cellular requirement for NLRP3 activation and the structure of NLRP3 have revealed the complex regulation of NLRP3 and the multiple steps involved in its activation. This review presents a perspective on the biochemical and cellular processes controlling the assembly of the NLRP3 inflammasome with particular emphasis on structural regulation and the role of organelles. We also highlight the latest research on metabolic control of this inflammatory pathway and discuss promising clinical targets for intervention.

## Introduction

A vital function of the immune system is to detect alterations in tissue homeostasis and respond in a context-dependent manner. To achieve this, immune and stromal cells, particularly those located at barrier sites, express a diverse range of germline-encoded receptors termed pattern recognition receptors (PRRs). These receptors detect foreign pathogen-derived molecular patterns or endogenously derived danger-associated molecular patterns (DAMPs) that signal pathogen invasion or tissue damage. The resulting signals from PRRs are processed and integrated to produce an inflammatory response that initiates an immune response to restore tissue homeostasis.

An essential family of PRRs is the inflammasome sensor family, cytosolic proteins that upon activation trigger inflammatory signaling and cell death. Inflammasomes are multiprotein complexes that consist of a sensor, the adaptor protein ASC, and caspase-1; these components assemble following sensor activation and oligomerization [1]. This complex enables the activation of caspase-1, which then cleaves and activates proinflammatory cytokines, including IL-1β, and gasdermin D (GSDMD), which can trigger pyroptosis, a type of lytic cell death [2]. Uniquely among PRRs, some inflammasome sensors do not directly bind pathogen-derived molecules but rather have evolved to detect perturbations in essential cellular systems [3]. This ability is advantageous for germline-encoded receptors, as it enables the detection of a diverse range of pathogens. However, these sensors are also more likely to be aberrantly activated under sterile conditions that trigger cellular stress similar to that initiated by pathogens.

The most well-characterized cell stress-responsive inflammasome is the NLRP3 inflammasome, which consists of a pyrin domain (PYD), a NACHT domain required for nucleotide binding with ATPase activity, and a leucine-rich repeat (LRR) domain [4]. Activation of NLRP3 is considered a two-step process, with an initial priming step required for increased expression of NLRP3 [5], followed by an activating stimulus step that elicits inflammasome formation. This second step is triggered by a range of both pathogen- and danger-associated molecules, including ATP and lysosome-disrupting crystalline substances such as silica, cholesterol, and monosodium urate (MSU) crystals, as well as by changes to glycolysis or the mitochondrial electron transport chain (ETC) [4]. In murine in vitro models of disease, the first step in NLRP3 inflammasome activation is typically achieved by priming the cells with a cytokine receptor or TLR ligand such as LPS, and the second step is then triggered by a molecule such as ATP, depending on the disease context. It is important to note that under some conditions, such as in human monocyte disease models, LPS alone can trigger NLRP3-mediated IL-1β release, which is discussed in more detail below. The diversity of NLRP3 stimuli indicates that NLRP3 acts as a key detection and response mechanism for cellular stress and suggests that NLRP3 monitors a system central to cellular function. The exact cellular processes monitored by NLRP3 are still not fully understood, but both mitochondria and, more recently, the trans-Golgi network (TGN) have been associated with NLRP3 activation [6]. Given the high propensity of NLRP3 to cause disease when aberrantly activated, it is not surprising that NLRP3 is also extensively regulated by posttranslational modifications (PTMs), including phosphorylation and ubiquitination.

While NLRP3 is essential for the immune response to numerous pathogens, it has primarily gained notoriety due to its role in autoinflammatory and inflammation-based diseases, including type II diabetes, Alzheimer’s disease (AD), atherosclerosis, and gout [7]. Mutations in NLRP3 cause autoinflammatory cryopyrin-associated periodic syndromes (CAPS) [8]. Therefore, it is a highly relevant target for therapeutic intervention, and indeed, multiple NLRP3 compounds are currently being evaluated in clinical trials [9].

In this review, we highlight the recent findings on NLRP3 activation with a particular focus on the relationship between inflammasome structure and its location upon activation. We also discuss the intricate processes required for full assembly and activation of the NLRP3 inflammasome and the promising therapies under development to modulate this inflammatory complex.

## NLRP3 molecular activation and organelle dysfunction

Despite its importance in infections and sterile tissue damage, the exact mechanisms controlling and enabling NLRP3 activation are still being elucidated. Diverse cellular perturbations trigger NLRP3 activation, including the disruption of cellular ion homeostasis, lysosomes, and mitochondrial function or metabolism. However, how these perturbations relate to each other and how they converge on a common molecular mechanism that activates NLRP3 remain ongoing areas of research. Here, we discuss how these factors may control the activation of NLRP3, focusing on recent insights into the spatial and structural regulation of this inflammasome.

### Potassium

Potassium efflux has been proposed as a general upstream requirement for activating the NLRP3 inflammasome. Many NLRP3 activators, including the potassium ionophores nigericin and gramicidin and pore-forming toxins that permeabilize the plasma membrane to potassium, enable potassium efflux [10–12]. Similarly, extracellular ATP-mediated opening of the P2X purinoceptor 7 (P2X7) channel and the two-pore domain weakly inward rectifying potassium channel 2 (TWIK2) also trigger potassium efflux [13, 14]. The involvement of potassium also holds true for particulate activators, including cholesterol crystals, silica, and calcium pyrophosphate crystals, which induce potassium efflux before NLRP3 activation. Notably, the dependence of NLRP3 activation on potassium efflux is usually determined by increasing the extracellular potassium concentration, which in all cases inhibits NLRP3 inflammasome activation. Perhaps the best demonstration of the dependence of the NLRP3 inflammasome on potassium efflux is the observation that potassium-deficient media is sufficient to trigger NLRP3 inflammasome activation in murine bone marrow-derived macrophages (BMDMs) [15]. However, while sufficient to activate NLRP3, potassium efflux is not an absolute requirement for NLRP3 activation by all factors. Recent studies have demonstrated that NLRP3 activation can also be potassium-independent. Imiquimod and its related compound CL097 activate NLRP3 but do not trigger potassium efflux [16]. Similarly, the alternative inflammasome pathway, present in human monocytes stimulated with LPS alone, is potassium efflux-independent and instead activates NLRP3 through a TLR4-TRIF-RIPK1-FADD-CASP8-dependent cascade [17]. These results suggest that some pathways may bypass potassium efflux, and consequently, further studies are required to elucidate the precise conditions in which potassium efflux is necessary to facilitate NLRP3 activation. Furthermore, the reason why potassium efflux is an activation trigger for NLRP3 is not understood at the molecular level.

### Chloride

Similar to the case for potassium, chloride efflux can play an essential role in NLRP3 inflammasome assembly. Chloride intracellular channel protein 1 (CLIC1) and 4 (CLIC4) have been reported to be required for NLRP3 activation by controlling chloride efflux. These channels translocate from the nucleus to the plasma membrane during LPS priming, and their ablation attenuates ATP-mediated NLRP3 activation [18]. A separate study similarly found a role for CLICs in NLRP3 activation and determined that potassium efflux promotes CLIC-dependent chloride efflux for optimal NLRP3 activation [19]. The kinase WNK-1 also regulates NLRP3 activation by controlling chloride flux. WNK-1 indirectly governs the activity of chloride/cation cotransporters, which limits chloride efflux and NLRP3 activation [20]. When potassium and chloride were investigated separately, the results showed that chloride efflux alone leads to the formation of inactive NLRP3-dependent ASC oligomers. Subsequent potassium efflux was required for caspase-1 cleavage and IL-1β release, suggesting that chloride efflux may enable ASC association and oligomerization but is not sufficient for forming a functional inflammasome [21]. Notably, chloride efflux is an early event in NLRP3 activation, occurring within the first 30 minutes [19], which may explain why this event was not observed in some studies [15]. Further investigations are required to delineate the relationship between chloride efflux and its effects on NLRP3 activation.

### Calcium

Cytosolic calcium flux has also been implicated in NLRP3 activation, as it was suggested to occur in response to NLRP3 stimuli, including alum, MSU, and nigericin [22–24]. Calcium enters the cell from either the extracellular space or calcium stores in the ER and Golgi. While calcium is essential for many cellular processes [25], disruption of calcium homeostasis affects several organelles, leading to ER and mitochondrial stress [26]. However, in contrast to earlier studies, more recent systematic studies demonstrated that calcium flux did not occur in response to most NLRP3 activators and, furthermore, that calcium flux is neither necessary nor sufficient for NLRP3 activation [15, 27]. Instead, the effect of calcium on many diverse cellular processes may underlie its considerable influence on NLRP3 activation. Indeed, calcium influx into mitochondria has been proposed to exacerbate mitochondrial stress and potentiate NLRP3 activation [24]. Furthermore, P2X7 facilitates sodium and calcium influx that, in turn, modulates TWIK2 to enhance potassium efflux [13], which together trigger NLRP3 activation [22]. The calcium-sensing receptor (CaSR) has also been described as a regulator of NLRP3 activation through its control of calcium flux and cAMP [23].

### Mitochondria

**Fig. 1:**
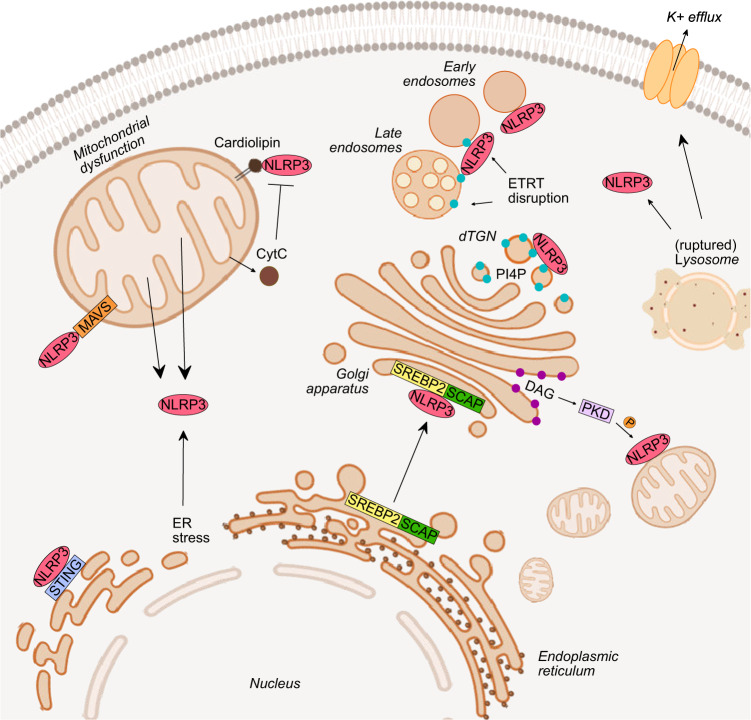
NLRP3 cellular localization. The cellular localization of NLRP3 has a significant impact on its activity and effector function. NLRP3 has been found in close proximity to mitochondria, the endoplasmic reticulum, and the Golgi apparatus. Changes to and within these organelles can directly contribute to NLRP3 activation, although the reciprocal effect of NLRP3 on these structures remains to be fully established

Mitochondrial dysfunction has been linked to NLRP3 inflammasome activation through the release of mitochondrial DNA (mtDNA) and mitochondrial ROS (mtROS) into the cytoplasm, as well as through its potential role as a platform for NLRP3 inflammasome assembly (Fig. 1). MtDNA was identified as a potent immunostimulatory molecule that acts on multiple PRRs to trigger a proinflammatory response [28]. The release of mtDNA into the cytosol occurs during apoptosis, which requires BAD and BAX signaling, as well as in response to a wide variety of cell stress triggers [20, 29], including NLRP3 activators such as ATP, hexokinase (HK) displacement from the mitochondria and nigericin [30, 31]. The exact mechanism of mtDNA release remains to be fully elucidated but has been suggested to occur in response to oxidative stress and to require the voltage-dependent anion channel [32]. Importantly, Shimada et al. showed that mtDNA must first be oxidized (ox-mtDNA) to activate NLRP3 [30]. This finding was supported by a subsequent study that demonstrated that unoxidized mtDNA, in contrast to ox-mtDNA, preferentially activates the inflammasome sensor AIM2 [33]. It has also been suggested that LPS-driven mtDNA synthesis is required for NLRP3 activation, as NLRP3 activation was inhibited by ablation of mtDNA polymerase (pol-g) and transcription factor A, mitochondrial (TFAM), which regulates mtDNA replication and integrity [33]. These studies found that ox-mtDNA interacts directly with NLRP3, but it is unclear whether this interaction is required for NLRP3 activation [30, 33]. Indeed, it was recently demonstrated that ongoing mitochondrial ATP production via the ETC is necessary for NLRP3 inflammasome activation [34]. As the ablation of mtDNA also disrupts the ETC, these data provide an alternative explanation for the requirement for both pol-g and TFAM for NLRP3 activation.

NLRP3 activation has also been suggested to be driven by mtROS, which are generated by complex III and, to a lesser extent, complex I of the ETC in the mitochondria, and the levels of mtROS can increase in response to cellular stress or ETC perturbation [35]. The NLRP3 activators ATP and MSU crystals were shown to trigger mtROS [36], as was imiquimod, which generates ROS by inhibiting both complex I of the ETC and the quinone oxidoreductase NQO2 [16]. Treatment with antioxidants prevents activation of the NLRP3 inflammasome by these stimuli, suggesting that mtROS are required. However, given the wide-ranging effects of antioxidants, further evidence is needed to confirm or refute the role of mtROS. Indeed, preliminary studies suggest that antioxidants inhibit NLRP3 priming rather than NLRP3 activation [37]. Recently, an elegant experiment by Billingham et al. addressed the requirement for mtROS in NLRP3 activation [34]. These authors reconstituted BMDMs deficient in ETC complex III activity with an alternative oxidase that enables electron flow and ATP generation but does not produce ROS. As anticipated, these macrophages showed reduced mtROS production without ETC disruption. Despite the lack of mtROS production, these cells displayed NLRP3 activation similar to that of wild-type cells, and the corresponding mouse strains had similar IL-1β levels when challenged with LPS in vivo. This is perhaps the most conclusive experiment examining the role of mtROS in NLRP3 activation and suggests that mtROS may be generated by NLRP3 activators but not causative of NLRP3 activation.

Mitophagy has also been suggested to regulate NLRP3 activation, as the degradation of dysfunctional or damaged mitochondria prevents the generation or release of mtROS and mtDNA. This was first demonstrated in BMDMs lacking general autophagy components; these cells accumulated damaged mitochondria and showed increased NLRP3 activation [38]. However, autophagy was also shown to limit the protein levels of NLRP3 and inflammasome activation [39, 40]. Subsequently, Zhong et al. used Park2-deficient BMDMs to ablate mitophagy without altering other autophagy pathways and found that explicitly preventing mitophagy enhances NLRP3 activation by a range of NLRP3 activators, including ATP, nigericin, and lysosomal disruption. These results collectively demonstrate that preventing the removal of damaged mitochondria enhances NLRP3 activation, suggesting that mitochondria are indeed involved in NLRP3 activation.

Mitochondria and associated mitochondrial membranes have been proposed to act as scaffolds for the localization of NLRP3 and the formation of the NLRP3 inflammasome. In unstimulated cells, NLRP3 is predominantly localized in the cytosol, but binding to a scaffold at a specific subcellular compartment is suggested to be required to support nucleation and inflammasome formation. Three different ligands on the surface of mitochondria have been suggested to bind and enable NLRP3 inflammasome activation: cardiolipin, mitochondrial antiviral signaling protein (MAVS), and mitofusin-2 [41–43]. Cardiolipin is a strongly negatively charged lipid that normally localizes to the inner mitochondrial membrane. Nevertheless, it is externalized in response to cell stress, where it is an essential scaffold for autophagy and the cell death apparatus [44]. Cardiolipin was shown to directly interact with NLRP3 through its LRR domain in the context of mitochondrial dysfunction, thereby driving NLRP3 activation [41]. A subsequent study demonstrated that LPS triggers cardiolipin binding to caspase-1, suggesting that it may function as a scaffold for NLRP3 activation [45].

In contrast, cytosolic release of the mitochondrial protein cytochrome c may play a regulatory role by competing with NLRP3 for binding to cardiolipin [46]. A reduction in cardiolipin synthesis was shown to reduce NLRP3 inflammasome activation [41]. Comparatively, the requirement for both MAVS and mitofusin-2 was specific to NLRP3 activation by RNA viruses [42, 43]. This localization to MAVS is dependent on the N-terminus of NLRP3 and is necessary for the optimal NLRP3 response [42]. More recently, NLRP3 was shown to assemble on the TGN rather than mitochondria [47], so further research is necessary to investigate the relevance of these pathways to NLRP3 activation.

### Lysosomal damage

Lysosomal damage or disruption is a crucial mechanism of NLRP3 inflammasome activation under many pathological conditions (Fig. 1) that can be triggered by the phagocytosis of either endogenous particulates, including MSU crystals, cholesterol- or deoxysphingoplipid-based lipid crystals and amyloid-β aggregates, or exogenous particulates, including silica, asbestos, and alum [48–52]. Phagocytosed crystals accumulate in the lysosomal compartment, causing increased lysosomal acidification, lysosomal swelling, a loss of lysosomal membrane integrity and, ultimately, NLRP3 inflammasome activation [48, 52]. NLRP3 activation is suggested to involve the release of cathepsins from the lysosome, as broad-spectrum cathepsin inhibitors prevent NLRP3 activation by lysosomal disruptors [52, 53]. However, BMDMs prepared from mice lacking one of cathepsin B, S, L, C, or X and from those lacking all five cathepsins showed no alteration or merely a minor decrease in NLRP3 inflammasome activation [53].

Furthermore, cathepsin inhibitors still blocked NLRP3 inflammasome activation in the combination cathepsin knockout, suggesting these compounds have cathepsin-independent targets or that there is further redundancy in the cathepsins that are still expressed. Notably, lysosomal damage-mediated NLRP3 activation is also dependent on potassium efflux, suggesting that lysosomal damage triggers permeabilization of the plasma membrane to potassium [48, 52]. Interestingly, lysosomal disruption leading to NLRP3 inflammasome activation can also be triggered by stimulator of interferon genes (STING) activation in human monocytes in response to dsDNA [54]. The relevance of this pathway is still unclear as it is human-specific. Nevertheless, the involvement of this pathway is intriguing, as it suggests that endogenous processes can control lysosomal stability, disrupting this stability in response to specific stress signals activates NLRP3.

### Endoplasmic reticulum

The ER has been associated with NLRP3 in several ways, including ER stress, the regulation of cholesterol homeostasis, and NLRP3 localization. Compound-induced ER stress can cause potassium efflux and the generation of ROS, thus triggering NLRP3-dependent IL-1β release. Interestingly, the unfolded protein response, another critical stress response pathway in the ER, does not play a role [55]. Another study showed that ER stress can also drive mitochondrial dysfunction in an NLRP3-dependent manner, leading to a “feed-forward loop” and subsequent NLRP3 activation [56]. A previous report demonstrated that NLRP3 partially colocalizes with the ER in unstimulated THP-1 cells. This localization changed in response to NLRP3 stimuli, causing NLRP3 to colocalize with ER and mitochondrial markers, predominantly in the perinuclear space [57].

The ER is the primary organelle for the regulation of cholesterol homeostasis. Cholesterol homeostasis is essential for cellular function, as cholesterol is an integral part of lipid rafts on the plasma membrane and lipid-rich regions in intracellular membranes. As such, cholesterol has been shown to regulate signaling hubs in cellular membranes for several critical pathways [58, 59]. Alterations in cholesterol homeostasis have also been associated with dysregulation of the inflammatory response, leading to altered signaling from immune receptors and the accumulation of intracellular cholesterol. Notably, the cholesterol content of the ER was proposed as a possible regulatory step in NLRP3 activation. Niemann-Pick disease, type C1 (NPC1) is a membrane protein that facilitates the egress of cholesterol from late endosomes and lysosomes into the cytosol. Inhibition of NPC1 through both pharmacological and genetic means reduces NLRP3 activation due to the decrease in cholesterol flow into the cytoplasm [60]. To determine if this effect is due to cholesterol depletion in the ER or the plasma membrane, de la Roche et al. used cholesterol-deficient media and brief statin treatment before NLRP3 activation to inhibit cholesterol biosynthesis and thus semi-selectively deplete the ER of its cholesterol content and found that this decrease in cholesterol accounted for the decreased NLRP3 activation [60].

Furthermore, this effect could be reversed by supplementing cells with lipid products downstream of HMG-CoA reductase, the target of statins. Together, these findings imply a connection between ER cholesterol content and NLRP3 activation. Nevertheless, given the wide-ranging effects of manipulating cellular cholesterol, further studies are required to fully understand the underlying mechanisms.

Interestingly, SREBP2, a master transcription factor for cholesterol homeostasis, has been linked to NLRP3 activation (Fig. 1). This relation is independent of its control of cholesterol homeostasis but relates instead to the interaction of SREBP2 with SCAP and their subsequent translocation from the ER to the Golgi [61]. NLRP3 was shown to directly interact with both SCAP and SREBP2 through its NACHT domain, and obstruction of this translocation leads to impaired NLRP3 activation, suggesting that both SREBP2 and SCAP control NLRP3 localization and modulate its activity [61]. Furthermore, another study demonstrated a distinct cGAS-STING-NLRP3 pathway in response to herpes simplex virus 1. In this context, the ER localization of NLRP3 was enhanced via its interaction with STING, leading to the deubiquitination of NLRP3 and its subsequent activation [62].

### The Golgi apparatus

The Golgi apparatus has recently been a focus point in deciphering the mechanism of NLRP3 activation. In addition to the previously described connection involving the translocation of SCAP and SREBP2 to the Golgi and their direct interaction with NLRP3, another important interaction partner was shown to be protein kinase D (PKD). NLRP3 stimuli evoke the enrichment of diacylglycerol (DAG) in Golgi membranes, resulting in PKD recruitment. NLRP3 stimuli also trigger the clustering of mitochondria and mitochondria-associated endoplasmic reticulum membranes (MAMs) around the Golgi, facilitating the phosphorylation of associated NLRP3 at S293 by PKD. This event leads to the release of NLRP3 from MAMs, which is required for the formation of the cytosolic NLRP3 inflammasome [63]. A more direct connection between NLRP3 activation and the Golgi was recently proposed based on a study showing that NLRP3 relocalizes to the dispersed TGN (dTGN). Diverse NLRP3 stimuli trigger reorganization of the trans face of the Golgi, which transitions from a perinuclear, compact structure to a dispersed network, coinciding with the distribution of NLRP3 puncta. Furthermore, Chen et al. demonstrated that the recruitment of NLRP3 to the dTGN is controlled by the positively charged polybasic region between the PYD and the NACHT domain of NLRP3 (residues 127–146), which enables the interaction of NLRP3 with phosphatidylinositol-4-phosphate (PI4P). Interestingly, in contrast to previous studies, mitochondrial localization was not observed in this study [47].

Subsequent studies investigating the regulation of NLRP3 recruitment to the dTGN showed that this process can be influenced by both Bruton’s tyrosine kinase (BTK) and inhibitor of nuclear factor kappa-B kinase subunit beta (IKKβ). BTK, which is required for NLRP3 activation through its kinase activity [64, 65], was subsequently shown to directly phosphorylate several tyrosine residues in the polybasic region of NLRP3, resulting in charge reversal of this region. This change leads to the disassociation of NLRP3 from PI4P, which is hypothesized to enable NLRP3 activation [66]. Similarly, pharmacological and genetic suppression of IKKβ prevents the interaction between NLRP3 and the TGN and inhibits NLRP3 activation. However, the residue(s) phosphorylated by IKKβ to facilitate this interaction has yet to be defined [67].

Investigation into the spatial dynamics of NLRP3 highlighted glycogen synthase kinase 3β (GSK3β) as an essential regulator of NLRP3 activation. GSK3β driven phosphorylation of phosphatidylinositol-4-kinase 2 Α (PI4k2A, an important kinase for the generation of PI4P, which regulates vesicular trafficking among the Golgi, endosomes, and lysosomes) controls the recruitment of NLRP3 to mitochondria and the Golgi. Interestingly, by tracking NLRP3 temporally and spatially, Arumugam et al. determined that NLRP3 first colocalizes with mitochondria, peaking at approximately 10–15 min post stimulation [68]. This is followed by a decrease in mitochondrial localization and a concomitant increase in Golgi localization [68]. In a different approach, Hong et al. used brefeldin A (BFA) to directly disrupt ER–Golgi trafficking and Golgi integrity and found that this attenuated NLRP3 activation [63, 69]. Knockdown of the primary target of BFA, guanine nucleotide exchange protein (BIG1), appears to have the same effect, whilst Golgi integrity was seemingly undisrupted. However, it is unclear whether the observed effects are due to inhibition of LPS-triggered priming [69]. Although this study suggests a role for ER–Golgi trafficking, further characterization of Golgi integrity and impaired priming are required to fully define the impact on NLRP3 activity.

### Vesicular trafficking and the endocytic network

The endosomal network is involved in many signaling pathways, acting as a scaffold for signaling complexes, regulating the localization of plasma membrane and endosomal receptors, and controlling both the uptake and delivery of signaling molecules from the environment [70]. Several articles recently submitted to preprint servers suggest that the endosomal network also regulates NLRP3 activation (Fig. 1). These studies suggest a more complex nature for dTGN dispersal. Specifically, they found that the TGN markers used in previous studies do not reside at the TGN but rather are trafficked between the TGN and the endosomal network through endosome-TGN retrograde transport (ETRT). Consequently, the authors of these recent studies propose that the dTGN observed in previous studies resulted from a block in endosomal trafficking in response to the disruption of retrograde transport between the endosomes and the TGN [71, 72]. Genetic disruption of ETRT by targeting critical regulators ADP-ribosylation factor-related protein 1(ARFRP1) and SYS1 evokes LPS-induced NLRP3 activation without requiring a second stimulus and potentiates NLRP3 activation through several common stimuli. Furthermore, Zhang et al. found that disruption of ETRT leads to PI4P accumulation and increases NLRP3 localization on endosomes [71], supporting the observations by Lee et al. [72].

Similarly, it has been proposed that NLRP3 acts as a sensor of defective endosome trafficking. Lee et al. demonstrated that several NLRP3 stimuli, such as nigericin, LeuLeu-OMe, and imiquimod, disrupt multiple trafficking pathways. Interestingly, the bona fide disruptor of intracellular trafficking monensin did not cause NLRP3 activation but did enhance IL-1β release, ASC speck formation, and cell death when combined with imiquimod, which originally had the mildest effect on intracellular trafficking among the tested stimuli. Notably, these researchers also demonstrated that NLRP3 causes PI4P accumulation on endosomes and NLRP3 localization [72]. Endosomal localization of NLRP3 is also vital for its activation by internalized membrane attack complex (MAC). In the resting state, NLRP3 was shown to predominantly colocalize with the ER and subsequently translocate to Rab5+ endosomes upon stimulation with MAC to facilitate inflammasome assembly. Interestingly, NLRP3 translocation to Rab5+ endosomes was not observed in response to ATP stimulation, suggesting that this effect is specific to activation by internalized MAC [73].

Altogether, in addition to ionic imbalance, organellar dysfunction appears to be an emerging common stimulus of NLRP3 activation. NLRP3 has been reported to localize to various organelles in response to different stimuli in different systems. Recent studies on the roles of the Golgi and the endosomal network, as well as previous studies describing the involvement of mitochondria, lysosomes, and ER, reveal a complex spatial and temporal component to the molecular mystery behind NLRP3 activation. Although the necessity for strict regulation may underlie this complexity, it is also possible that a portion of the cellular pool of NLRP3 acts as a sentinel around one or many organelles. As with other PRRs, NLRP3 needs to be in the “right place” to sense the relevant molecular triggers for activation. The recent findings proposing the Golgi and possibly the endosomal network as sites of NLRP3 recruitment represent the most recent advancements in the field, yet further studies are required to completely elucidate the spatial regulation of NLRP3.

## Structure–function relationship for NLRP3

Until recently, information on the structural arrangement of NLRP3 has been lacking, largely due to technical difficulties in using structural assays to analyze NLRP3. However, recent studies examining the structure of NLRP3 in complex with NEK7 and its inactive oligomeric form have provided new insights into its structure and how structure may regulate function. Given the difficulties with working with full-length NLRP3, early structural studies instead focused on the effector PYD of NLRP3 in X-ray crystallography and NMR. These studies revealed the similarity of the PYD to that of its NLR structural homologs (NLRP1/4/7/10/12); this domain contains six helices of varying lengths and orientations among different NLRs [74], and the NLRP3 PYD and ASC PYD self-associate through homotypic protein regions [75].

Next, new insight was provided by the crystal structure of NLRP3 in complex with the mitotic kinase NEK7. NEK7, a member of the NIMA-related kinase family, was identified as a direct interaction partner of NLRP3, and its binding to NLRP3 was shown to be required for NLRP3 activation in murine macrophages [76–78]. Notably, this NEK7 dependency does not seem to extend to the human system in the same manner [79]. The interaction of NEK7 with NLRP3 was subsequently proposed to enable NLRP3 oligomerization [80]. Structural analysis of the NEK7-NLRP3 crystal structure revealed that NEK7 interacts with the NACHT and LRR domains of NLRP3. Mutations in NEK7 and NLRP3 that disrupt the interaction interface lead to decreased NLRP3 activation in a reconstituted murine macrophage cell lines [80]. The receptor for activated protein C kinase 1 (RACK1) was also shown to be an interaction partner for both NLRP3 and NEK7 [81]. Knockdown of RACK1 strongly inhibits NLRP3 activation in BMDMs by reducing its ability to form an oligomer with NEK7. Furthermore, studies using bioluminescence resonance energy transfer (BRET) to distinguish the conformation of NLRP3 showed that ablation of RACK1 attenuates the transition of NLRP3 from its “closed” resting state into an “open” conformation in response to nigericin.

The conformational changes in NLRP3 during activation have also been studied utilizing a BRET-based NLRP3 sensor. Compan et al. found that this method could be used to distinguish the “open” and “closed” states of NLRP3 [82]. NLRP3 stimulation leads to a decreased BRET signal as NLRP3 transitions into a more “open” conformation [83]. Studies utilizing this tool showed that potassium efflux promotes NLRP3 to adopt an “open” conformation [83]. This conformational change requires the region between the PYD and NACHT domain, including a linker region (aa 91–132) and a short domain named FISNA﻿ (fish-specific NACHT associated, aa 135–208). Notably, deletions in the linker region (delta 92–132) inhibit NLRP3 activation, which is fully abolished by further deletions into the FISNA domain [83]. Moreover, replacement of the PYD and linker of NLRP6 (an NLR that does not respond to potassium efflux-dependent stimuli) with the PYD, linker, and FISNA domains of NLRP3 render the chimera protein sensitive to potassium efflux [83]. Intriguingly, the potassium-independent stimulus imiquimod also evokes an “open” NLRP3 conformation, suggesting that this conformational transition is not entirely dependent on potassium [83]. Thus, the precise conditions that facilitate conformational changes remain to be elucidated.

A recent development in the biochemical understanding of NLRP3 came from the characterization of the structure of the inactive NLRP3 oligomer or “cage”. Two independent studies described oligomers for human and mouse NLRP3 in either an ATP-bound conformation or an ADP-bound form with or without the NLRP3 inhibitor CP-456,773 (also known as CRID3 or MCC950), which stabilizes NLRP3 in its inactive conformation [84, 85]. Notably, human NLRP3 forms a 10-mer, while mouse NLRP3 forms 12- to 16-mers. Both oligomers adopt a double-ring structure that seems to shield the PYD [84, 85]. In both species, oligomer formation requires LRR–LRR interactions between two monomers, and these pairs then interact with other pairs through back-to-back LRR interactions to form a double ring [84, 85]. Mutations in the interfaces that regulate LRR–LRR interactions in the human NLRP3 cage lead to autoactive and hyperactive NLRP3, as shown in an ASC speck formation assay, corroborating their inhibitory role [85]. Although the overall configurations of the human and mouse oligomers are comparable, distinct differences exist regarding interfaces and stoichiometry, most likely due to a combination of technical variance and species-specific divergence.

Interestingly, two previous studies determined that the double-ring cages are membrane-bound, supporting data from cell-based studies [84, 85]. Further spatial investigations into mouse NLRP3 cages revealed that they regulate TGN localization, as mutations in NLRP3 that affect double-ring cage formation decrease TGN dispersal [84]. This finding implies that the NLRP3 cage is not onlycrucial for localization to the TGN but also regulates TGN dispersal. Defective cage formation was also prompted by mutations in the previously reported polybasic region of NLRP3, leading to impaired TGN relocation [84]. Interestingly, artificially directing this mutated NLRP3 to the TGN using an oxysterol-binding protein 1 tag re-establishes cage formation, suggesting that membrane association may be a requisite first step in cage assembly [84].

In summary, it is clear in the context of both murine and human cells that NLRP3 requires a scaffold for activation, such as NEK7. Whether this is now the exhaustive list or there are other factors required is still unclear. The structure of the inactive form of NLRP3, along with biochemical studies demonstrating that NLRP3 transitions from one state to another when stimulated, and that this transition can be blocked by CP-456,773, suggests that NLRP3 must undergo a conformational change in order to be activated. This transition is also suggested to be linked with the requirement for low potassium through the FISNA domain. How this transition occurs and how subsequent NLRP3 activation proceeds are open questions. Further studies are also required to decipher the cellular roles of the NLRP3 cage in the context of its localization, identify all the interaction partners, and ascertain the function of PTMs.

## Posttranslational modifications controlling NLRP3 inflammasome activation

**Fig. 2:**
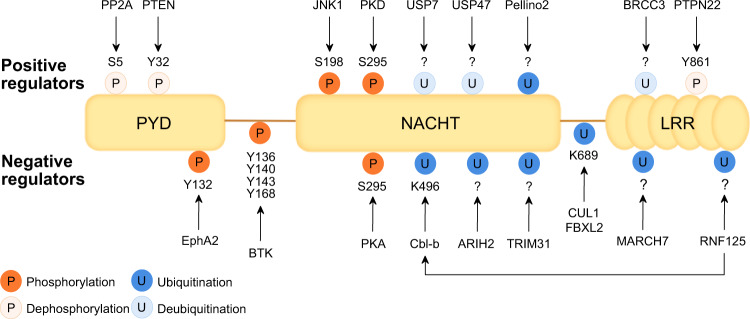
Posttranslational modification (PTM) of NLRP3. NLRP3 activity is controlled by various PTMs, including the ubiquitination of lysine residues and the phosphorylation of serine and tyrosine residues, which either enhance (top panel) or reduce (lower panel) NLRP3 assembly and activity. PTMs are regulated by a variety of enzymes, and the regulation of these enzymes may represent therapeutic strategies for diseases involving NLRP3

The innate immune system relies on multilayered mechanisms that regulate the intensity and duration of responses. One of the most dynamic mechanisms is the PTMs of a protein, which can change its structure, localization, and activity by conjugating a chemical group or a small protein to an amino acid within a protein. PRR signaling is subject to extensive regulation by PTMs targeting the sensors themselves and their downstream adaptors and effector molecules [86, 87]. Several PTMs of NLRP3 have been identified that contribute to the complexity of its activation and inflammasome formation (Fig. 2). Here, we focus on the most extensively studied PTMs related to NLRP3, namely, ubiquitination and phosphorylation. Other NLRP3 PTMs and modifications associated with ASC, caspase-1, and its substrates have been reviewed elsewhere [88, 89].

### Ubiquitination/deubiquitination

Ubiquitination, the first PTM reported on NLRP3 [90], consists of the attachment of ubiquitin to lysine residues via isopeptide linkages catalyzed by E3 ubiquitin ligases [87]. Ubiquitination of NLRP3, through the addition of either K48-linked or K63-linked ubiquitin chains, negatively regulates different steps of inflammasome activation either by targeting NLRP3 for proteasomal degradation or by preventing protein‒protein interactions.

Under basal conditions, E3 ubiquitin ligases contribute to maintaining low levels of NLRP3. For instance, F-box/LRR-repeat protein 2 binds NLRP3 at W73 and leads to its degradation through ubiquitination of K689 [91]. Tripartite motif-containing protein 31 (TRIM31) also limits NLRP3 abundance through the K48 ubiquitin‒proteasome pathway. LPS or IL-1β stimulation increases cytosolic TRIM31 levels, suggesting that this E3 ligase plays a role in a feedback loop that downregulates inflammasome activation [92]. NLRP3 ubiquitination and degradation also occurs in the nervous system, where the neurotransmitter dopamine functions as an endogenous inhibitor in a mechanism dependent on MARCH7-mediated K48 polyubiquitination in the LRR domain [39]. Upon stimulation, NLRP3 needs to be licensed for activation. Cullin1 (CUL1) associates with NLRP3 to promote K63 ubiquitination at K689, which blocks the interaction between NLRP3 and ASC, preventing inflammasome assembly. Inflammasome activators induce CUL1 dissociation from NLRP3 to initiate the signaling cascade [93]. Ariadne homolog 2 (ARIH2) has also been shown to negatively regulate inflammasome assembly. Interestingly, genetic ablation of ARIH2 completely abolishes NLRP3 ubiquitination in the NACHT domain and promotes inflammasome activation, while its overexpression enhances K48 and K63 modification and suppresses activation [94].

During priming, the LRR domain is subjected to K63 polyubiquitination mediated by RING finger protein 125. This modification induces the binding of casitas-B-lineage lymphoma protein-b, which promotes NLRP3 degradation by K48 polyubiquitination at K496 within the NACHT domain [95]. Pellino2 also catalyzes the K63 polyubiquitination of NLRP3 in the priming phase to facilitate NLRP3 activation by subsequent activating stimuli, but the precise mechanism remains unknown [96]. In the nontranscriptional priming of NLRP3, TNFR-associated factor 6 favors its oligomerization and interaction with ASC via K63 ubiquitination, although the modification site has not yet been determined [97].

Deubiquitination is suggested to be necessary for full NLRP3 activation. This modification may be involved in the rapid activation of NLRP3, which can be triggered after a short priming time (30 min or less) [98]. After LPS or Pam3CSK4 exposure, Abraxas brother protein 1 binds NLRP3 phosphorylated at S198 by c-Jun N-terminal kinase (JNK). This binding event, in turn, recruits the BRCC3-containing BRISC complex, which removes ubiquitin chains from the LRR domain, permitting NLRP3 to interact with ASC [90, 99]. Moreover, knockdown of ubiquitin-specific peptidase (USP) 7 and USP47 was shown to decrease ASC speck formation and inflammasome activation, although the exact underlying mechanisms are still unclear [100].

### Phosphorylation/dephosphorylation

PTM of NLRP3 by phosphorylation occurs by adding a phosphate group to serine or tyrosine residues. Phosphorylation plays a predominant role in regulating NLRP3 oligomerization and localization and orchestrates signaling networks required for NLRP3 activation.

In the resting state, unintended oligomerization of NLRP3 is prevented by the AKT-induced phosphorylation of S5, which hinders interactions with the PYD of NLRP3 via electrostatic repulsion. Dephosphorylation of S5 by phosphatase 2A (PP2A) licenses NLRP3 for downstream signaling, but subsequent stimuli are required to fully assemble the inflammasome [101, 102]. Recently, TBK1 and IKKε were found to cooperate to control AKT and ultimately counteract PP2A to limit NLRP3 activity. However, TBK1 and IKKε also target site(s) other than S5, suggesting that the effects are not restricted to AKT [103]. TGF-β-activated kinase 1 (TAK1) may also be important for avoiding spontaneous NLRP3 activation, as in the absence of TLR priming and second signals, TAK1 deficiency induces NLRP3 inflammasome activation in macrophages [104]. TAK1 is thought to restrict NLRP3 activation by preventing aberrant RIPK1-dependent activation of NF-κB and extracellular signal-related kinase and subsequent initiation of cell death pathways. In the LRR domain, Y861 is phosphorylated by an unknown kinase, leading to autophagy-dependent NLRP3 degradation. Upon exposure to ATP or MSU crystals, protein tyrosine phosphatase non-receptor 22 directly dephosphorylates Y861, impeding NLRP3 recruitment to phagophores and positively regulating IL-1β secretion [105, 106].

As mentioned above, stimulation of TLRs in the early phase of priming induces NLRP3 phosphorylation at S198 by JNK1 to promote BRCC3-mediated deubiquitination of NLRP3 and oligomerization. JNK1-deficient macrophages and those expressing an S198A mutant completely fail to activate the NLRP3 inflammasome, and the same effect was observed in a mouse model of CAPS treated with JNK1 inhibitors [107]. Similarly, phosphorylation of S803 by CSNK1A1 during priming controls the recruitment of NEK7 to NLRP3, which is another prerequisite for BRCC3 binding and deubiquitination of NLRP3 [108]. Positive regulation of the NLRP3 inflammasome is mediated by phosphatase and tensin homolog (PTEN), a well-known tumor suppressor. PTEN directly interacts with NLRP3 and mediates dephosphorylation of Y32 in the PYD, enabling the NLRP3–ASC interaction and inflammasome maturation. It has been suggested that PTEN determines chemotherapy sensitivity by promoting NLRP3-dependent antitumor responses in myeloid cells [109]. In the NACHT domain, S295 undergoes phosphorylation by protein kinase A (PKA) in response to stimulation by bile acids or prostaglandin E_2_ signaling. Mechanistically, PKA reduces NLRP3 ATPase activity, which is critical for oligomerization, thereby inhibiting inflammasome formation. Interestingly, S295 phosphorylation is impaired in CAPS-mutant NLRP3 [110, 111].

An essential focus of NLRP3 inflammasome research is the movement of components across subcellular compartments. One PTM that is known to control this movement is PKD-mediated phosphorylation of NLRP3 at S295. In response to activating stimuli, MAMs localize adjacent to the Golgi, where DAG levels rapidly increase, promoting PKD activity and subsequent NLRP3 release from MAMs into the cytoplasm, leading to inflammasome assembly [63]. Similarly, BTK also phosphorylates NLRP3 at Y136, Y140, Y143, and Y168 in the PYD-NACHT polybasic linker. These modifications alter the charge of the linker domain from net positive to net negative, leading to a shift in NLRP3 localization from intact to dispersed Golgi membranes and promoting oligomerization, interaction with ASC, and IL-1β release. Regulation of kinase activity offers a possible strategy for the development of therapeutics, as inhibition of BTK blocks both NLRP3 inflammasome formation and IL-1β cytokine release [66].

Overall, PTMs play essential roles in regulating the activation cycle of the NLRP3 inflammasome. However, future research is required to explore the interplay among various PTMs to elucidate how they cooperate to influence NLRP3 activation and inflammasome assembly. Indeed, the enzymes responsible for modifying NLRP3 are attractive therapeutic targets for numerous inflammation-driven diseases that are attributed to dysregulated NLRP3 activation.

### NLRP3 and cell death

Genetically programmed cell death is fundamental for maintaining tissue homeostasis in both developing and adult organisms, as well as for tumor suppression, defense against infection, and prevention of autoimmunity and sterile inflammation in the host [112]. The most well-described cell death pathways are apoptosis, necroptosis, and pyroptosis. Apoptosis is generally considered an immunologically silent form of cell death, as there is no release of cytosolic contents into the extracellular space. Conversely, necroptosis and pyroptosis result in plasma membrane rupture that mediates the release of various DAMPs, which, as discussed above, are immunogenic potentiators of inflammatory responses [113–115]. Notably, the NLRP3 inflammasome is intimately intertwined with all of these forms of cell death, as the formation of this inflammasome occurs downstream of the initiation of cell death and is governed by apoptotic and necroptotic signaling molecules [116]. Considering the importance of NLRP3 in pathological conditions, a better understanding of the regulation and crosstalk of programmed cell death with NLRP3 activation is vital for the development of therapeutic interventions. This section will focus on how cell death pathways initiate NLRP3 inflammasome activation downstream of disruption of plasma membrane integrity and on the caspase-8-mediated control of NLRP3 inflammasome activation.

As described in previous sections, the mechanisms and molecules that trigger cytoplasmic potassium efflux are potent activators of NLRP3. Notably, key cell death effector molecules, including the necroptotic effector molecule mixed lineage kinase domain-like protein (MLKL), the pyroptotic effector GSDMD, and cleavage and opening of the plasma membrane channel pannexin-1, permeabilize the plasma membrane to ions, enabling potassium efflux [117]. This potassium efflux then triggers NLRP3 inflammasome activation, leading to the cleavage and release of proinflammatory cytokines, including IL-1β, thus causing inflammatory cell death.

GSDMD is a critical molecule in the execution of pyroptosis. GSDMD activation depends on its cleavage, which releases the N-terminal fragment and leads to oligomerization, translocation, and subsequent perforation of the plasma membrane [118]. Caspase-1/-4/-5/-11 were the first caspases identified to cleave GSDMD, enabling pore formation. Direct interaction with endotoxin results in caspase self-activation, subsequent pannexin-1 cleavage, potassium efflux, and NLRP3 inflammasome activation [119]. Consequently, GSDMD was initially characterized as an effector of pyroptotic death [120, 121]. However, it was subsequently determined that caspase-8 can also directly cleave and activate GSDMD under specific circumstances, which consequently triggers activation of the NLRP3 inflammasome due to disruption of the intracellular ionic balance [122, 123]. In addition to GSDMD, other members of the gasdermin protein family, such as GSDMA3, GSDMB, and GSDME, can also execute pyroptosis-like death and therefore also trigger NLRP3 induction downstream of apoptotic signaling and proteolysis of the abovementioned gasdermins by caspase-3/-6/-7 [124, 125]. Activation of NLRP3 downstream of gasdermin cleavage thus enables caspases to trigger inflammation and cell death.

The plasma membrane glycoprotein pannexin-1 is also a crucial regulator of NLRP3 activation downstream of cell death. Proteolysis of pannexin-1 by both apoptotic and inflammatory caspases at its autoinhibitory C-terminus results in channel opening, subsequent permeabilization of the plasma membrane to ions, and potassium efflux [126]. Pannexin-1 cleavage was first shown to play a role in the activation of NLRP3 in noncanonical inflammasome activation, in which its cleavage by murine caspase-11 (or caspase-4/-5 in humans) is required alongside GSDMD cleavage [127]. Subsequently, it was determined that activating both extrinsic and intrinsic apoptosis can similarly trigger NLRP3 inflammasome activation through cleavage of pannexin-1 by the executioner caspases (3/6/7) downstream of either caspase-8 or caspase-9 [128]. Interestingly, these findings challenge the concept that apoptosis is immunologically silent, as NLRP3 activation downstream of apoptosis will trigger inflammation. One area of application of such a finding is in cancer chemotherapeutics designed to trigger cell death by activating the apoptosis regulators Bax and Bak, which elicit IL-1β secretion during the late stages of apoptosis, most likely through caspase-3/-7-mediated pannexin-1 cleavage [129]. In addition, SMAC-mimetics and TAK1 inhibitors trigger caspase-8 activation, which can also cleave and open pannexin-1, similarly engaging the NLRP3 inflammasome [130]. Consequently, while apoptotic cell death remains immunologically silent under homeostatic conditions and does not involve IL-1β secretion, prior exposure of cells to inflammatory stimuli that trigger increased expression of NLRP3 enables cancer and inflammatory therapeutics to drive NLRP3 activation and apoptosis downstream of caspase-8/-9 activation. This is relevant to cancer immunotherapy, as several studies have proposed that acute IL-1β signaling is essential for antitumor immunity [131, 132]. Together, these studies implicate secondary NLRP3 inflammasome activation in circumstances of prolonged apoptotic signaling, inefficient removal of apoptotic bodies, or defective clearance of apoptotic cells [117, 133]. Consequently, the NLRP3 inflammasome has the capacity to alter apoptotic cell death signaling to a more proinflammatory process, depending on cell type, the potency of the signal received, and the inflammatory status of the environment prior to receiving the apoptotic stimulus.

Necroptosis is a form of cell death initiated to protect against pathogen infection or cellular damage. It is characterized by cell swelling, membrane rupture, and the release of DAMPs, which subsequently mediate inflammation. Necroptotic signaling is triggered by ligation to death receptors, such as TNF receptor, Fas, TLR3, TLR4, and ZBP1 [134], which activate the kinase RIP1 in response to the inhibition or absence of caspase-8. RIPK1 then recruits and activates receptor-interacting serine/threonine-protein kinase 3 (RIPK3), which phosphorylates the pseudokinase MLKL, causing it to oligomerize and insert into the plasma membrane, consequently disrupting plasma membrane integrity [114]. To date, many studies have demonstrated that the NLRP3 inflammasome is activated downstream of MLKL pore formation and that this activation is independent of GSDMD [135, 136]; examples include activation of TLR3 by dsRNA and activation of TNFR in the presence of caspase-8 inhibition [137]. The physiological importance of NLRP3 activation downstream of necroptotic signaling is relevant for the inflammatory response to bacterial and viral infection, as both viruses and bacteria often encode caspase inhibitors [138]. This importance is highlighted by the reported finding that RIPK3-MLKL-mediated inflammasome activation is crucial to restraining *Salmonella* infection in the intestinal mucosa; specifically, MLKL^-/-^ mice show impaired caspase-1 cleavage and pathogen clearance [139]. In contrast, *Staphylococcus aureus* toxin-induced infection is associated with exacerbated inflammation due to NLRP3 engagement downstream of MLKL activation [140]. Other experiments in which NLRP3 inflammasome activity was inhibited downstream of MLKL-induced inflammation demonstrated beneficial effects for necroptotic and MLKL-driven diseases [141]. Thus, further studies are necessary to delineate the exact contribution of NLRP3 and IL-1β signaling to MLKL-dependent pathologies, as targeting components of the NLRP3 pathway may represent therapeutic strategies.

As mentioned previously, caspase-8 is a key apoptotic molecule that has a well-appreciated role as an initiator caspase during extrinsic apoptosis signaling. In addition, caspase-8 has been demonstrated to be a central regulator of NLRP3, for which it has both positive and negative regulatory functions [142]. Negative regulation of NLRP3 by caspase-8 is seen in the context of caspase-8 deficiency, and restriction of caspase-8 activity is known to result in excessive inflammation due to uncontrolled necroptosis, which, as mentioned previously, is a potent NLRP3 stimulus [143, 144]. However, surprisingly, caspase-8 deficiency was found to be sufficient to activate the NLRP3 inflammasome in dendritic cells and macrophages independent of necroptosis [145]. Instead, the loss of NLRP3 restriction under caspase-8-deficient conditions involves RIP3 and the downstream necrotic signaling molecules MLKL and phosphoglycerate mutase/protein phosphatase 5 (PGMA5) [145]. Notably, RIP3-dependent, necroptosis-independent NLRP3 activation requires inhibitor of apoptosis proteins (IAPs), as the activation of NLRP3 upon the loss of both IAP and caspase-8 requires MLKL-mediated plasma membrane permeabilization [146]. However, other mechanisms through which caspase-8 restricts NLRP3 activation remain to be elucidated.

Caspase-8 can also induce NLRP3 inflammasome formation under certain circumstances [147]. In murine BMDM, engagement of TLR4 in the absence of IAPs or intracellular infection with *influenza* virus results in RIPK3-dependent caspase-8 activation and subsequent NLRP3 inflammasome induction, which is independent of MLKL [146, 148]. Similarly, TLR4 activation in human monocytes can directly activate NLRP3 through a process referred to as the alternative inflammasome. In this process, NLRP3 is activated by TLR4-TRIF-RIPK1-FADD-CASP8 in a manner independent of potassium efflux, pyroptosome formation, and cell death [17]. Of note, recent studies also identified apolipoprotein C3 as a signal for alternative inflammasome formation [149]. By uncoupling inflammasome activation from lytic cell death, alternative inflammasome activation consequently provides a platform for sustained inflammatory signaling that may provide adequate defense against pathogen invasion.

The exact mechanism by which caspase-8 activates the NLRP3-caspase-1 axis remains to be determined. Direct cleavage of NLRP3 in alternative inflammasome activation has been ruled out, but proteolysis of an unidentified signaling mediator is a possible scenario. Independent of its enzymatic function, caspase-8 can drive NLRP3 assembly by its scaffolding function during dsRNA signaling via TLR3 [137]. Furthermore, canonical and noncanonical inflammasome priming has been reported to be governed by caspase-8 and FADD [150]. At the posttranslational level, both active caspase-8 and its enzymatically inactive homolog cFLIP_L_ have been reported to interact with NLRP3 and thereby drive its activation and caspase-1 processing [150, 151].

Taken together, data indicate that NLRP3 inflammasome activation downstream of membrane permeabilizing events and of the inhibition of innate signaling or caspase-8 appears to be an effective mechanism to support and amplify immune responses and thus contributes to pathogen clearance and protection against infection. The interplay among and flexibility of cell death pathways have likely evolved as compensatory mechanisms to avoid harm to the organism, as perturbations in specific effector molecules in cell death pathways by pathogenic proteins, such as TAK1 by YopJ and caspase-8 by viral FLIP proteins, are rapidly recognized by the host cell, which in turn mounts a response against the invading pathogen [122]. Further research is needed to establish how cooperation among these genetically and biochemically distinct cell death pathways either contributes to other pathophysiology or might be beneficial for health; such data may improve our understanding of prospective anticancer and anti-inflammatory therapeutic strategies.

These findings show that NLRP3 inflammasome activation is intertwined with apoptotic, necroptotic, and pyroptotic cell death. Its activation downstream of plasma membrane permeabilization occurs upon cleavage and activation of cell death effector molecules, such as pannexin-1, MLKL, and members of the gasdermin family. Consequently, depending on the cell type and inflammatory environment, the NLRP3 inflammasome can shape apoptotic signaling to a more proinflammatory process. In addition to potassium efflux, the NLRP3-caspase-1 axis is also regulated by caspase-8; however, the exact mechanism of action remains to be elucidated.

## Regulation of the NLRP3 inflammasome by metabolism

While we have discussed how mitochondria are closely linked to NLRP3 activation and inflammasome assembly, these organelles are best known as the powerhouse of the cell due to their ability to generate energy in the form of ATP. However, the nutrients used to fuel mitochondria significantly impact cellular function. During inflammatory stimulation, cells utilize glycolysis to quickly break down glucose and rapidly produce ATP to power cellular activity. However, mechanisms such as oxidative phosphorylation are preferentially used during basal or anti-inflammatory conditions. Recent developments have revealed that the metabolites generated as part of these processes play a central role in fine-tuning cellular activity, extending to modulating or enhancing the activity of the NLRP3 inflammasome and its components.

NLRP3 activation can be modulated or triggered by different enzymes in the glycolytic pathway. The first step of glycolysis is controlled by hexokinase-1 (HK1), which phosphorylates glucose, controlling its entry into this pathway. However, the absence of HK1 attenuates LPS and ATP induced NLRP3 inflammasome activation with a significant reduction in the release of IL-1β, IL-18, and cleaved caspase-1 [152]. In line with these findings, the inhibition of glycolysis by 2-deoxy-D-glucose prevents NLRP3 inflammasome activation, thus blocking the production of IL-1β in vivo [153] and IL-1β, IL-18, and mtROS in vitro [152, 154]. HK also detects N-acetyl glucosamine (a component of bacterial peptidoglycan), triggering dissociation from the mitochondria and activating the NLRP3 inflammasome [31]. Pyruvate kinase isozyme M2 (PKM2) catalyzes the final step in glycolysis, leading to the formation of pyruvate and ATP. Xie et al. demonstrated that PKM2 regulates the release of IL-1β, IL-18, and cleaved caspase-1 from macrophages via the PKM2-dependent phosphorylation of EIF2AK2 [155]. In contrast to the approach in these reports, other studies have blocked glycolysis after glucose entry by inhibiting either GAPDH or α-enolase and shown that this triggers NLRP3 inflammasome activation [156]. This activation was due to a related drop in NADH levels and an increase in mtROS, highlighting that glycolysis can regulate NLRP3 in different ways. Further research is required to more completely understand these complex processes.

Several glycolytic intermediates are associated with enhanced NLRP3 inflammasome signaling. Upon stimulation with LPS, macrophages undergo a switch to glycolysis that increases the levels of the metabolite succinate. Succinate stabilizes the transcription factor HIF-1α [154], which in turn increases the production of IL-1β while reducing that of IL-1RA [154, 157]. This process is mediated by succinate dehydrogenase (SDH); preventing succinate oxidation by the administration of dimethyl malonate (an inhibitor of SDH) reduces HIF-1α, thus impairing the production of IL-1β while enhancing that of IL-1RA [157]. Succinate also increases ROS production [157], and as discussed above, mtROS has been implicated in NLRP3 activation [57]. Activation of the NLRP3 inflammasome can, in turn, feedback onto these pathways; specifically, IL-1β can further increase glycolysis in macrophages, elevating the expression of the glycolytic enzyme PFKFB3 [158].

While few studies have examined these pathways in microglia, it has been found that LPS increases glycolysis in these CNS-resident myeloid cells and induces an increase in pro-IL-1β expression by stabilizing HIF-1α [159]. In addition, microglia in murine AD models have increased NLRP3 inflammasome activation [160] and enhanced glycolytic activity [161].

Innate immune cells have also developed a sophisticated system to resolve inflammation after stimulation. Along with its effects on succinate, LPS also increases the levels of the metabolite itaconate by increasing the expression of immunoresponsive gene 1 (*Irg1*), which encodes the enzyme that produces itaconate through the decarboxylation of cis-aconitate [162]. Itaconate is an endogenous inhibitor of SDH [163, 164], and its accumulation leads to a reduction in HIF-1α and mtROS [164].

Itaconate also directly regulates the NLRP3 inflammasome, reducing the mRNA levels of many inflammasome components, including *Pycard, Casp1, Il1b*, and *IL18*, and attenuating the NLRP3 inflammasome-induced cleavage and release of IL-1β [164]. Indeed, *Irg1*^-/-^ macrophages show enhanced IL-1β release in response to NLRP3 inflammasome stimuli [165]. In line with this, a recent report found that itaconate tolerizes macrophages and impairs late NLRP3 inflammasome activation in LPS-primed cells [166]. The itaconate derivative 4-octyl-itaconate (4-OI) reduces glycolysis by modifying the glycolytic enzyme GAPDH [167] and protects against LPS-induced IL-1β secretion by human and murine macrophages, reducing the levels of HIF-1α and mtROS [168]. 4-OI can also regulate NLRP3 inflammasome activation, preventing the interaction of NLRP3 with NEK7 by modifying C548 on NLRP3 [165]. 4-OI can similarly reduce LPS-triggered death and the release of IL-1β in vivo [167]. It should be noted that these effects are restricted to the 4-OI derivative of itaconate, and there is some controversy about whether itaconate works through the same mechanism [169].

Fumarate is another metabolite that can directly affect inflammasome signaling. A recent report by Humphries et al. demonstrated that fumarate can block NLRP3 inflammasome-induced cell death and IL-1β release by modifying the cysteine residues of GSDMD to S-(2-succinyl)-cysteine, resulting in its inhibition. These modifications prevent GSDMD from interacting with caspase-1, and in vivo studies found that fumarate protects mice from LPS-induced IL-1β in vivo and reduces disease severity in a murine model of multiple sclerosis [170]. In line with this finding, fumarate can reduce IL-1β production in microglia in vitro and in vivo and alleviate LPS-induced sickness behavior in mice [171, 172].

Ketone bodies also modulate the NLRP3 inflammasome. β-Hydroxybutyrate (BHB) is a ketone produced in the liver that can be used as an energy source in animals on a low-carbohydrate diet (the so-called ketogenic diet) or under conditions of nutrient deprivation. BHB efficiently blocks the NLRP3-induced formation of ASC specks and prevents the consequent production of cleaved caspase-1 and IL-1β [173]. Interestingly, AD patients have lower circulating levels of BHB [174, 175], and treatment of transgenic AD models with BHB reduces the levels of NLRP3 inflammasome-dependent ASC specks, cleaved caspase-1, and IL-1β while attenuating Aβ-plaque deposition [175]. In line with this, several AD clinical trials have found that chronic intake of a ketogenic formula or adherence to a ketogenic diet improves cognition in dietary-compliant individuals. Nevertheless, the effects were reduced in the washout period [176, 177].

Together, these findings highlight the crucial role that metabolites can play in controlling the NLRP3-mediated immune response. Changes in glycolysis and increasing levels of succinate can enhance NLRP3-mediated inflammation and prolong inflammatory signaling. In contrast, shifting the balance of metabolites to increase itaconate or fumarate can restrict inflammasome activation and IL-1β release. Further research on these pathways may provide insight into alternative ways to therapeutically regulate NLRP3 via the direct targeting of metabolites and metabolic signaling.

## Therapeutic targeting

Aberrant NLRP3-driven inflammation underpins many chronic degenerative diseases, including type II diabetes, gout, atherosclerosis, inflammatory bowel disease, and AD [7]. Therefore, NLRP3 inhibition represents a potential therapeutic strategy for many of these conditions, and the development of an NLRP3 inhibitor has been pursued vigorously in recent years. Compounds that have been developed thus far and have entered clinical trials include IFM-2427 (IFM/Novartis), Somalix (Inflazome), dapansutrile (Olatec), and NT-0167 (Nodthera) [9]. Of the available compounds and strategies to inhibit NLRP3, those that directly inhibit the NLRP3 inflammasome by binding to the NACHT domain of NLRP3 are expected to be the most specific.

These approaches have been discussed extensively in other reviews [9, 178], so this section will instead focus on the implications of recent findings related to the structure of NLRP3 in drug development. As mentioned earlier, two recently published cryo-EM structures of inactive NLRP3 demonstrate that NLRP3 is held in an inactive form with a decamer cage-like conformation in humans [85] or a double cage-like conformation of 12-mer to 16-mer in mice [84]. The decametric cage conformation requires the interaction of an acidic loop in the LRR with a basic patch in an adjacent LRR. Mutation of the acidic loop leads to hyperactivation of NLRP3, suggesting that this structure forms before NLRP3 activation. Notably, both structures were solved in the presence of the NLRP3 inhibitor CP-456,773 and ADP, and the results showed that CP-456,773 binds to NLRP3 near the ATP-binding Walker A motif site. This compound interacts with five separate subdomains, stabilizing the inactive conformation of the NACHT domain [85]. As such, CP-456,773 likely prevents the conformational change required to bind ATP, thus preventing NLRP3 activation. This finding is consistent with previous findings demonstrating that CP-456,773 inhibits ATP binding to NLRP3 and affects the conformational change upon activation [179, 180]. These data suggest that compounds targeting this site stabilize the inactive, decametric form of NLRP3 to prevent its activation and may provide further information for the design or alteration of NLRP3 inhibitors.

Further information from clinical trials of NLRP3-targeting compounds should provide insight into the efficacy of targeting NLRP3 for the treatment or prevention of NLRP3-related diseases, while the NLRP3 structure highlights an effective target to prevent NLRP3 inflammasome activation.

## Outlook

The spatial regulation and licensing of NLRP3 rely on a highly dynamic network of intracellular organelles and interaction partners, facilitating the maturation of this protein into the active form. In addition, the temporal order of the described events remains elusive in many cases; along with the relative dispensability of specific pathways, this alludes to a complex web of sequential and separate pathways that lead to a saturation of mature activatable NLRP3 to achieve inflammasome assembly. Furthermore, species-, cell type- and stimuli-dependent variations create another layer to unravel. As much of the work discussed in this review was performed in macrophages, the extent to which these complex mechanisms occur in other cell types and disease states remains to be established. NLRP3 appears to localize to various organelles under different conditions in its transition toward a functional inflammasome. However, the recent appreciation of Golgi and endosome involvement provides exciting avenues for further research. The elucidation of these pathways will highlight the points at which these diverse stimuli converge, forming a unifying model for NLRP3 activation.

Even though the relationship between activation and structure remains to be further explored, structural studies provide a basis for developing drugs and inhibitors for therapeutic and research purposes. The recent discovery of NLRP3 cages creates a new and exciting avenue for this work. While the proposed membrane localization of NLRP3 remains to be further explored, the combination of spatial and structural research on NLRP3 will undoubtedly considerably impact future studies.
